
JOURNAL INFORMATION
==============================
NLM Title Abbreviation: Med Chir Rev
NLM Journal ID: 0034617

The Medico-Chirurgical Review

Publisher: Biomedical Journal Digitization Project

ARTICLE INFORMATION
==============================
PMCID: PMC4966180
PMID: 29917603
Article version: 1
Subjects: Articles

Periscope, or Circumspective Review

Print publication date: 1828 Jul 1
Volume: 9
Issue: 17
First page: 145
Last page: 157
1828 Apr 12
License: This work is free of known copyright restrictions. For more information, please see PMC Back Issue Digitization.
License URL: https://creativecommons.org/publicdomain/mark/1.0/

==============================

### Periscope;

OR,

### CIRCUMSPECTIVE REVIEW.

"Ore trahit quodcunque potest, atque addit acervo."

[12th APRIL, 1828.]

1. Traumatic Tetanus.—Mr. Abernethy.

This distinguished lecturer, after describing the symptoms of tetanus, "which seems to be chiefly a muscular affection" —takes notice of the great insensibility of the nervous system—remarks that "alkohol will not fuddle a tetanic patient; opium will not make him sleep; mercury will not salivate him." This last observation is incorrect. Tetanic patients are very frequently salivated. On coming to the treatment, the lecturer displays great erudition. "Hippocrates has told us almost as much about this disease as we have known ever since."

"He says that, in certain cases of tetanus, where the fits increase, and there are exacerbations of the symptoms, the patient generally dies on a *particular day* —I do not exactly remember on which, but not far distant from the commencement of the disease."—*Lectures, p.* 22.

If Hippocrates had been able to tell the particular day on which a tetanic patient would die, he would have told us much more than the subsequent experience of 2000 years has enabled us to ascertain. It is a pity that the worthy lecturer did not refresh his memory by a peep into the works of Hippocrates, before he attributed to the Father of Physic such wonderful sagacity. This *particular day* is as follows:—"Hic, tertia die, aut quinta, aut septima, aut decimaquarta perit." *De morbis*, III. XIII. That the sly old man of Cos has, like Dr. Moore in his prognostications, left himself pretty many loop-holes to creep through, in case of necessity, must be obvious enough. That a tetanic patient may happen to die on one of those days enumerated within the fortnight, is very probable—but that he is just as likely to die on one of any other four days within the same period, we venture to assert.

Hippocrates has gone a little farther than our present knowledge warrants. He says, if the patient survives the 14th day, he will recover. This is not the case. In respect to treatment, the lecturer tells us that Hippocrates recommends the cold affusion as the *most effectual* remedy in tetanus, not traumatic. But the fact is, that the Father of Physic makes this the *very last item* in his methodus medendi.

"Huic catapotia ex pipere et veratro nigro devoranda dato: et jusculum volucrium pingue calidum. Inducendæ sunt etiam fortes ac multæ sternutationes et fomenta adhibenda." *ib.*

Finally he says:—

"Quod *si voles*, aquam frigidam plurimam superfundito, et postea vestimenta tenuia, pura, ac *calida* superintegito." *ib.*

Such are the directions of Hippocrates, lauded by Mr. Abernethy, as "confirmed during the progress of medical science," and, in truth, comprehending, "all that is known with regard to the treatment of this disease."

It is no wonder that medical science should be over-run with ERROR, when errors, like these, are taught at the very fountain of knowledge!

"I know (says Mr. A.) of no specific remedy for tetanus. The application of the cold affusion seems to be only administering to a symptom. It lessens muscular action, (query,) at least for a time, because whatever lessens the temperature of the body, lessens muscular action."

But Mr. A. does not tell us that the temperature of the body is preternaturally increased in tetanus—indeed he could not do so with justice. And is it true that *muscular action* is decreased by the application of cold ? We hardly have seen a more contradictory and inconsistent medley of observations than are portrayed in this lecture on tetanus. After the eulogy on Hippocrates, who recommended the black pepper and cold affusion, we were surprised to find that Mr. Abernethy had ventured to improve on a plan which was considered as embracing all that we knew of therapeutics in this formidable malady.

" I can only say, that we ought to pay especial attention to the removal of that irritation of the nervous system, which so constantly arises from a disordered state of the digestive organs. It is difficult to get the bowels to act in tetanus, but it is a most essential point to attend to their secretion. The abdominal muscles act with so much difficulty in this disease, that there is little fear of overdosing your patient. Give calomel and jalap with treacle, which will answer better than saline medicines. If a patient is delirious, or is so violent that he cannot be induced to take a dose of medicine, I watch an opportunity when he opens his mouth, put a gag into it, whip the medicine with a spatula over the tip of his tongue, and then take out the gag. I have purged patients in this way, when I knew not how I should have accomplished it in any other manner. When the calomel and jalap have been administered till they act on the bowels, they will at last generally bring away such stuff as people who have never seen it, can have little idea of. I remember, on one occasion, asking an old nurse what sort of evacuations had come from a tetanic patient, who had passed a week without having any, ' Lord, Sir, they are not stools—they are sloughs !' "

Mr. A. states that, in a patient who had taken large quantities of opium, 30 drachms of opium were found undissolved in the stomach. This may serve to shew that there is something in the stomachs of tetanic patients that prevents the operation, or even the solution of opium. How this man could have retained *solid* opium so long in his stomach, where there must have been fluids constantly in contact with it, is almost incomprehensible.

The lecturer notices the curious fact that tetanus sometimes comes on, when the wound has become clean, and the cicatrix rapidly advancing. The strength and the bias of *system* are well illustrated in the lecturer's mode of accounting for this untoward event. During the sloughing state of the wound the " digestive organs" become disordered, and this disorder produces tetanus when the wound is healing! If disorder of the stomach and bowels were capable of inducing tetanus, independent of wounds or other causes, we should soon have a thin population in this country!*

---

### 2. Refined Food and Education of Children.

Under the above head, Dr. Leonard Stewart has made some ingenious observations, in a little work " On Tendency to Disease," which, we think, are not undeserving of notice. The Doctor properly remarks, that the pliancy of the human constitution, in enduring various modes of living, though great, is not unlimited. It exists in greatest force from puberty to middle age—though its abuse often dates from an earlier period. Infancy is subject to epidemic and accidental diseases, as well as other periods—and, in refined life, children have to labour under the effects of hereditary weakness, and over-officious care.

" We see tender parents begin their toil of meddling and indulgence, by over exciting young people, and giving them food which is too fine and too nourishing. Articles of diet are sought out entirely freed from coarse admixture or dross ; these soon pass from the stomach, which eagerly calls for a fresh supply of such easily digested matter ; but, containing, in large proportion, the elements of chyle, they remain long exposed to the process of assimilation in the small intestines, where the absorbing lacteal vessels have an excess of work. The desired end of this

---

* We learn by a letter in the last Medical Gazette, that Mr. A. denies the *correctness* of these lectures, whether as published in the Lancet, or in the volume in question. It, therefore, becomes the more necessary to examine the doctrines and facts which have so long passed current, as those of Mr. Abernethy.

ill-judged plan of nourishment, that, namely, of fattening and strengthening, is at first very frequently attained ; but even this is a dangerous advantage ; for, the intestines being clogged, in consequence of the undue *remora* of the alimentary mass, the foundation is often laid of habitual constipation, plethora, nervous irritability, and, probably, a tendency to suffer from any accidental cause of disease. In other cases, nutrition is checked at its source by the excessive stimulation of the organs which are destined to effect it ; and, from congestion in the mesenteric region, glandular obstructions and marasmus are speedily induced. These causes operate with surer effect upon children from birth delicate, and who are kept from free exercise and the open air, either by constitutional inertness, or the short-sighted kindness of *mammas* and *grandmammas*."*

The intellectual appetite is not less pampered than the corporeal. Anxious to keep evil at a distance, and, if possible, to forestal the age of reason and reflection, instructors prematurely inspire caution and suspicion, and are much too exclusive in their choice of employment for the mind :—

"Substituting their own cleverness for the instinctive demands of the restless curiosity and thoughtless enterprise of youth (which, expending itself upon every object, leads to an early acquaintance with danger, and begets the habit of struggling with difficulty), they will have every thing select and safe ; and capriciously misdirect that spontaneous willingness which 'loves whate'er it looks upon,' and knows no care until it is suggested by more cunning heads."

It is impossible, as our author observes, to take Nature's work entirely out of her hands—at least with impunity. Yet, great pains are constantly taken to make "young people see with old eyes," and to "encumber them with help," when they should be allowed to grow and expand alone. "They are kept from falling instead of being taught to recover themselves when down."

"All this, however, is of trifling moment, when compared with the mischievous ingenuity with which early talent and precocious disposition is forced and overdeveloped. The spur is used where the rein should be. Learning is aimed at through some short cut ; pleasure is made easy and inviting : they come undiluted by dulness, unpurchased by labour—the rose without its thorn. The passions, eager for immediate gratification, will not endure the weariness of anticipation, and seize upon their bait ; but, too young and too undisciplined to contain or digest their fill of occupation, they soon fly off to some new object which, in its turn, they but taste. In this way, neither pleasure nor employment is fully proved or dwelt upon ; and the inclinations get a habit of fickleness and frivolity, which leads to general disgust. And when any of these half-tasted enjoyments is shown in the back ground of a long vista of toil and care, as the reward of him who pursues the path of industry, unused to difficulty, the enervated youth shrinks at the means of success, and imagining that he has tried every thing, he despises the end of exertion. Thus cloyed, but not gratified—sophisticated, but having no real knowledge—the spoiled children of weak, indulgent parents, exhaust the freshness of their halcyon existence ; and when the stubborn realities of after days are forced upon them, all the charm, all the *couleur de rose*, of life is gone !"

There are many observations contained in Dr. Stewart's work, which would be productive of much benefit to children, if they were generally distributed among the non-professional public.

---

### 3. Effects of Onanism on the Heart.

Dr. Krimer has lately published some melancholy cases, illustrating the effects of this destructive vice, on the central organ of the circulation. We shall adduce one or two cases, by way of example, and then give Dr. K.'s diagnostic marks of this class of complaints.

*Case* 1. A young man, aged 22 years, consulted Dr. K., presenting the following phenomena :—His constitution seemed to have been robust—complained of pain in the præcordial region, accompanied by cephalalgia, anxiety, and palpi-

* "On Tendency of Disease of Body and Mind."—p. 7—8.

tation. His tongue was clean, the eyes sunk, the edges of the eye-lids red, the countenance troubled and timid, the action of the heart very violent, the epigastrium sensible to the slightest pressure, appetite and bowels natural, pulse 85, full, but unequal. This young man had been living intemperately, and attributed his illness to over-exertion and fatigue, as a soldier. From certain evasive answers, and the general appearance of the patient, Dr. K. suspected onanism as the cause of the above symptoms, and delicately touched on the subject, portraying the terrible consequences which often result from that vice. The young man slunk away, without a single word of reply, and, for some months afterwards, carefully avoided the sight of the doctor. Dr. K., however, learnt that he became melancholy, taciturn, shunned society, especially the society of females, and spent the greater part of his time in reading medical books. Dr. K. went, uninvited, to visit this young man, and found him studying the "Manual of Beer." He presented a sad spectacle of pallor, debility, and anxiety. The heart was acting violently and irregularly, beating visibly over a considerable space, and audible at some distance from the patient. The epigastrium was still very tender to the touch. In this interview, the young man confessed his addiction to the vice in question, since the age of 14 years. Since the first interview, he had tried to break from the diabolical spell by which he was bound, but in vain. Dr. K. prescribed some sedative and antispasmodic medicines, and had the young man watched night and day for three weeks, to prevent a repetition of the vice. By these means the habit was broken, for a time, and the symptoms were greatly mitigated. But again he relapsed into his loathsome habits, and Dr. K. had recourse to a stratagem. By way of a local application, which was to act as a preventative, Dr. K. applied the caustic potash, which produced great ulceration of the prepuce, and this was kept up for several weeks. This effectually stopped the practice—the violent action of the heart subsided—and he ultimately got married, and continues to enjoy good health.

*Case* 2. A student in medicine, aged 21 years, had been addicted to this vice, since the age of fourteen. He was of feeble constitution, narrow chest, pallid countenance. He was propped up in bed when Dr. K. visited him, and appeared pale, dejected, his eyes sunken and haggard, with a surrounding blue circle, the head reclining forward on the chest, breathing short, action of the heart irregular, tumultuous, visible and audible—pulse full, hard, and 85 in the minute. There was heard, in the region of the heart, a tumultuous noise, like the boiling of water, or churning of a fluid. There was also a violent pulsation at the epigastrium, which was extremely sensible to pressure. He complained of head-ache, and had frequent attacks of epistaxis—pains in his arms and shoulders—nausea—bad digestion—in short, almost the whole catalogue of diseases to which humanity is heir! The Doctor candidly confesses, that he set down the complaint as enlargement of the heart, with sub-acute carditis, and treated it accordingly. But he soon found out his mistake, and, on close examination, the young man disclosed the real cause of this terrible commotion in the system. Two friends were appointed to sleep in the room with the patient, and watch him night and day, so as to prevent him from resorting to the baneful habits that had plunged him into this abyss of wretchedness. In order, however, to make more sure of obedience to his injunctions, he performed circumcision —the prepuce being naturally very long —and dressed the parts with irritating ointments, which effectually prevented all attempts at onanism. By this procedure, and some sedative medicines, the symptoms above-mentioned gradually subsided, and the young man was restored to health.

Some remarkable instances are detailed by our author, of this vice, in the other sex, but these we shall pass over. The following signs are considered by Dr. K. as diagnostic of cardiac affections resulting from onanism.

1mo. The hair is dry, not glossy, split at the extremities, and apt to fall off, especially from the fore-part of the head. In phthisical patients, and in those affected with real organic diseases of the heart, the hair is glossy and the reverse of the above.

2do. The eye is lack-lustre, hollow, watering, without expression, and often red at the edges of the eye-lids, with a surrounding circle of blue. It is the reverse in phthisis, and organic diseases of the heart.

3tio. The look is unsettled, timid, and the individual cannot bear the stedfast gaze of another person—a circumstance that is very characteristic.

4to. The head-ache is sometimes peculiar—inclining to periodicity—the pain radiating from the occiput to the frontal region.

5to. The sight is troubled or diminished—the appetite impaired—the tongue slightly furred—the breathing is often short, though the individual has the power of fully expanding the chest.

6to. There is almost continual pain in the stomach, with excessive sensibility at the epigastrium, but without the other symptoms indicative of inflammatory action in the mucous membrane of that organ. This pain, and this tenderness at the pit of the stomach, often exist where there is real organic disease of the heart, but they are by no means so constant.

7mo. There is great lassitude, with pain in the limbs, and especially about the loins and sacrum—disagreeable odour from the perspiration—somnolency—inaptitude for mental or corporeal exertion—great depression of spirits.

Such are the phenomena which characterise this affection of the heart, when arising from onanism, according to the experience of our author, who has very narrowly watched the symptoms in a very great number of cases. What the actual condition of the heart is, in such cases, he cannot tell, none having died of the complaint, and consequently no post-mortem examination having been made. There can be no doubt, however, that the disturbance of function in the organ of the circulation is purely a nervous affection, and unconnected necessarily with any change of structure. The disgusting nature of the subject has prevented English writers from any description or investigation of the phenomena; but we are well convinced, from many cases which have presented themselves to our observation, and where the cause has been voluntarily confessed, or unexpectedly drawn forth, that a great number of cardiac affections, as well as anomalous symptoms of disorder in other parts of the system, are owing to this destructive vice. We have, therefore, though reluctantly, been induced to draw the attention of our brethren to this melancholy item in the list of human failings, because it is, assuredly, a prolific source of misery—nay, of death.—HUFELAND'S JOURNAL, 1827.

---

### 4. INFLUENCE OF THE DIGESTIVE ORGANS ON THE MIND.

Mr. Cooke, in his recent publication, has not overlooked this curious and important subject, and, although he does not attempt the unravelling of such an intricate problem, he furnishes some cases in exemplification of the influence in question. We shall notice one of these cases.

A clergyman, of plethoric habit, consulted Mr. Cooke for what had been termed "determination to the head," and for which his reverence had been well bled and purged by a physician and surgeon, without the least relief. He had now become extremely irascible, and "his existence was quite oppressive to him." In his head he suffered extremely from a sense of tightness, heaviness, and irritation, without any vertigo, confusion of thought, or defect of memory. The tongue was furred, the bowels irregular, the excretions unhealthy. Alteratives, bitters, aperients, and a more generous diet were ordered—and antimoniated ointment was directed to be rubbed over the shaven scalp, as Mr. C. considered "that the disorder of the head consisted of a spasmodic affection of the occipito-frontalis muscle, and some of the muscles of the face." The patient improved in general health; but the contractions of the occipito-frontalis, &c. continued. He then took the carb. ferri, under which he farther improved. In a country village where he went for a few days, he was seized, while walking up an ascent, with an increase of the cerebral affection, by which he was much alarmed, and a medical gentleman who was called in, hearing the patient's vivid description of his sensations, considered the case as one of great urgency, if not danger. He proposed immediate phlebotomy, and—"when he saw the patient hesitating, he imprudently told him that, the pupils of his eyes were dilated, and he would not answer for his life *for five minutes*, if he were not bled." Bled he was—and blistered too—by which a temporary relief was afforded; but the strength was reduced, and the spirits more depressed. Mr. C. was summoned—prescribed aperients, and light tonics. "He gradually improved; but at the end of a fortnight returned home worse than when he left."

"The great imprudence of directing the mind of a morbidly susceptible man to a particular and very uncertain symptom as indicative of apoplexy, was often strikingly exemplified. He daily watched the pupils of his eyes, and soon he thought they appeared larger than usual. Though previously in a pretty comfortable state he became exceedingly alarmed, and the perturbation of mind reproduced throbbing of the head and a sense of confused intellect. I was sent for peremptorily, and preparation had been made in expectation that the lancet must be used, though he almost regarded it as signing his death warrant. He described the state of his head in the most pathetic terms—throbbing—giddy—confused—and almost every other formidable epithet was borrowed to define his sensations. His pulse, too, as might be expected, was rapid and strong. It was easy to perceive that his descriptions were exaggerated, and that the sensations he suffered were the result of nervous excitement. He was assured that there was no danger, and after soothing his fears and giving him a glass of wine, he soon became tranquil, and the affection of the head subsided without resorting to means of an exhausting nature."

We have often witnessed such instances as the above. While inditing this short article, a gentleman who had been reading a popular work "for the use of families," became convinced that he laboured under some disease of the heart, and repaired immediately to a physician in the city. The doctor, no doubt, found inordinate action of the organ, with every now and then an intermission of the pulse. He ordered a number of leeches to the region of the heart, and afterwards a blister. The patient slept none the following night, and on the third day, we saw him. The action of the heart was really tremendous, and while examining the chest by the stethoscope, the pulsations appeared ready to beat through the ribs. We immediately desisted, and made an excuse that we were obliged to write an answer to a note, during which the patient was requested to read a newspaper which was on the table. This apparent *sang froid*, on our part, had evidently some effect on the patient. We purposely prolonged the writing of the note, and in a quarter of an hour, the action of the heart was quiet and regular. He was told that, this time, he must pay a guinea for nothing—for he required no medicine—the leeching and blister had completely removed the disease. He buttoned up his waistcoat, and putting down the fee, declared he never paid one with greater pleasure in his life.

But to return to Mr. Cooke's patient. Having been sent to Brighton, he was there seized with a paroxysm similar to those already described, "and under his perturbation a physician, rather celebrated for his knowledge of *one form of cerebral disease* (query, who is this monomaniac medico?) was sent for, and immediately ordered cupping." The patient remonstrated against bleeding, which he was told would be injurious, when the Brighton physician "descended from his dignity, and applied to him epithets of vulgar abuse." Cupping, therefore, was employed and repeated, and the poor patient got worse and worse—in fact, "he was rapidly sinking, but by means of a post-chaise and four, he escaped once more to London. Mr. C. found him in a state of great depression, with quick pulse, white tongue, much debility. Bark and bitters, with gentle aperients were ordered. Under this treatment, aided by country air, and strict dietetics, he recovered health of body and tranquillity of mind.

There is no doubt that the rage for bleeding in all local demonstrations of chronic complaints does much harm. It is the same with drastic purgation in deranged states of the digestive organs. It is rather a wrong principle, however, to inculcate on the minds of irritable nervous patients, that "bleeding will never agree with their constitutions"—a notion which they are very tenacious of afterwards, and thus they embarrass their medical attendants when real inflammatory diseases occur. We see ridiculous instances of this very often. We do not, therefore, much wonder that the Brighton physician was worked into a rage at finding this pocket precept thrown in his face by the reverend patient.

5. Periodical Contraction of the Lower Extremities after Suppression of the Menses.

Dr. Fallot, a distinguished physician, at Namur, has published a curious case illustrative of the anomalous affections which often follow interruption of the catamenia.

*Case.* A strong country-girl, being then in the period of menstruation, was pursued by a dog, which she believed to be mad. She was greatly frightened, and the catamenia were suddenly suppressed. This was in May, 1826, and in December of the same year, she consulted Dr. F. there being no return of the uterine discharge, although leeches innumerable had been applied to the labia, &c. and semicupia had repeatedly been used. In the month of September, she began to experience a sence of troublesome formication in the lower extremities, attended with painful twitchings, which she could not prevent by any effort of the will. In October, these twitchings amounted to convulsive contractions of the legs, by which the heels were brought in approximation with the hips, and any attempt to stretch out the legs produced severe pain. This state of rigid contraction lasted five days, and then ceased. At the expiration of a month, the same phenomenon returned, lasted four or five days, and then vanished. In all other respects, the patient appeared to be in perfect health, being plump, ruddy, and strong. Dr. F. now conceived that the complaint depended on a periodical congestion in the vessels of the spinal marrow or its membranes—and therefore ordered 30 leeches on each side of the spine, at its lower extremity, between the loins and os coccygis. The flow of blood was abundant; but, three days afterwards, the contraction returned, though not in a violent degree. When the spasm vanished, the leeches were several times reiterated, in the course of the succeeding month, and the contraction returned no more. The menses, however, did not re-appear till the month of March, 1827. She now continues regular, and in the enjoyment of good health.—*Journ. Complem. Fev.* 1828.

6. Modes of Medical Practice.

In a report published in the Philadelphia Journal, by Dr. Jackson, there are some good remarks on the three principal plans of medical practice, now generally pursued in Europe and America,—viz: the Medicina Expectans—the Medicina Perturbatrix—and the Medicina Physiologica of Broussais. The First is the most ancient, and is founded on the fact, that the animal economy is capable of correcting, to a certain extent, deviations of the natural actions, constituting disease. We see even the most acute fevers and inflammations run a course—come to a crisis—and terminate favourably. In short, we every day see the recuperative powers of the constitution, were it not for which, the Doctor would make but a bad figure sometimes. This was the practice of the ancients, and is still influential on the Continent, especially in France, where the works of Hippocrates are in great esteem. It would probably be beneficial if a greater leaven of this were introduced into English practice.

The Medicina Perturbatrix reigns universally in the British Isles—and pretty generally throughout America. Here as there—"Nature is disclaimed as an auxiliary, and her efforts are regarded as more mischievous than beneficial. To use the language of Rush, Nature, when the physician enters the sick-room, is to be turned out of doors, as a noisy cat creating a disturbance."

In acting on this principle, the object is to cut short the disease, at once, and therefore, the most energetic means are employed. "The kingdom of heaven suffereth violence, and the violent take it by force." Matt. 2. This is a text, Dr. Jackson says, which is adapted to this school. Heroic remedies are "thrown in," with ceaseless activity—every organ is called into action—every function is disturbed—every emunctory is forced open, in order to drive through it the disease, as if it were an entity.

"Bleeding, vomiting, purging, sweating, urination, salivation, expectoration, stimulation, rapidly succeed each other, and not unfrequently are attempted all at one time; remedies become every thing; they are regarded as omnipotent; nature, the conservative powers of the system, are sunk into nothing, their sanative influence is unknown, or superciliously treated with contempt; though happily for the patient, those despised powers, often triumph over the disease, physic, and the doctor, combined to his injury."

There is a great deal of truth in the foregoing remarks, more especially as relates to chronic diseases. These heroic medicines are, in the highest degree, perturbating in their actions, and possess a degree of activity not inferior to any, and superior to most of the morbid causes by which diseases are generated. If this be true, we may form some idea of the mischief which is done by strong medicines in the hands of ignorance and temerity! We fear there is great room for reform in this important department of medical practice.

———— t.

### 7. Extensive Spinal Disease.

A young man, 26 years of age, born of phthisical parents, and who lost two sisters by pulmonary disease, took to the occupation of chimney-sweeper, at the age of twelve, and led a drunken and irregular life—sometimes sleeping on wet straw, in barns and out-houses, Summer and Winter. After a night spent in this way, he experienced some stiffness in the lower extremities, accompanied by weakness. These spread to the arms—and, in the course of a year he became affected with vertigo in the head, and tremor of the limbs. These incapacitated him for his occupation. One day he fell some yards down a chimney, and cut his head against the iron scraper; but the wound healed, without much difficulty. Nevertheless, the trembling, and weakness of the limbs, increased very much after this accident, and he was obliged to seek refuge in an hospital. When received, he presented the following symptoms:—tremor, and even convulsions, (on making any exertions) in the upper extremities—inability to stand or walk, without stumbling—heat and sensibility of the surface natural—no pain in any part of the spinal column—constant sense of vertigo—no head-ache—chest well formed—respiration easy and deep, with the power of lying in any position—all the secretions and excretions natural—appetite good—intellectual faculties weak.

The disease was considered to be, effusion into the vertebral canal, with some affection also of the head. A seton was made in the nucha, and various remedies were prescribed, which we shall not here enumerate. Moxas were also applied to the spine. A long, and pretty severe course of tartar-emetic, however, nearly removed the tremors and vertigo. The patient had got to the quantity of 24 grains of tartrite of antimony in the 24 hours. But the patient did not gain strength, and he left the hospital uncured. In July, 1826, he had another fall down a chimney—bruised his chest—and spat blood, with cough, pain in the thorax, and other symptoms which led the medical attendants to suspect serious mischief. A violent fever supervened, and again he entered the hospital. On examination, no disease of the lungs could be ascertained, but the heart was found pulsating in the right side of the chest. The expectoration indicated approaching phthisis, and was mixed with blood. After various vacillations, and a variety of remedies directed towards symptoms as they arose, anasarca came on, and the miserable patient sank exhausted, on the 16th November, 1826.

*Dissection.* There were several vesicles filled with fluid between the dura mater and pia mater—the latter membrane was every where thickened and opake—substance of the anterior lobes of the brain remarkably dense—six ounces of clear water in the ventricles—no disease of cerebellum. The spinal marrow was examined with great care. Nothing wrong appeared in its coverings. When these were slit open in front, the spinal marrow bulged out in five different places, giving the appearance of five salient points composed of whiter substance than the rest of the cord. On examining the two superior projections, the structure of the cord was found disorganized, and changed into a substance resembling thick pus. Beneath the third prominence there was found imbedded in the spinal cord, a solid body, of a kidney shape, and extremely vascular. A similar substance was discovered in the fourth projection. Between these salient parts, the spinal marrow presented a natural appearance. The lungs were disorganized—partly tuberculous, partly suppurated. The heart and pericardium were unaltered.—*Journ. Complem. Fev.* 1828.

We shall not enter into the numerous remarks and reflexions which the author has appended to this curious case. It is evident that great disorganization both of the brain and spinal marrow must have obtained, at the time when this miserable man was pursuing his avocation of chimney-sweeping, four or five months before his death!

---

### 8. Angina Pectoris.

Mr. Cooke, in his practical and very valuable Treatise on the "Digestive Organs," has made some observations on angina pectoris, which will be found to coincide with the opinions we have ventured to promulgate respecting this distressing complaint. Mr. C. remarks that this disease is, "perhaps *generally connected with ossification of the coronary arteries*, and a degeneracy of the muscular structure of the heart." With the latter part of the sentence we agree, but not quite so with the former. Indeed, we think, the following passage from Mr. Cooke, will negative the doctrine of coronary ossification.

"I have met with numerous instances of ossification in the coronary arteries which had never been attended with symptoms of angina: and cases of the latter have fallen under my notice where there was no ossification of the nutrient arteries, but there existed opaque depositions and other traces of disease at the root of the aorta and origins of the coronaries, *and likewise that flaccidity of the muscular structure which is usually found after this disease.* Therefore, it is not essential that ossification should have taken place, although ossific depositions are commonly found either in the course of the vessels or at their origins."

Mr. C. thinks that paroxysms of the disease are occasionally produced by derangement of the digestive organs, especially by dyspepsia. We shall give the particulars of a case related by our author at page 275 of his work.

*Case.* An Essex malster, aged 65 years, who had lived temperately and enjoyed good health, except being troubled, for some years, with flatulence and constipation, was seized, about three weeks before his death, with severe pain across the chest and down one arm, which he was unable to move, lest the motion should cause instant death. In a little time, the attack subsided, and he felt tolerably well. Three weeks after this first attack, he travelled outside of the coach to London, and, though well cloathed, he felt very cold. He was seized in Mark-lane-market, with pain in the epigastrium, extending beneath the sternum, for which he took some brandy, but without relief. He retired to a friend's house in White Chapel, where he complained of what he called "violent rheumatism of the left arm," especially about the wrist, connected with agonising pain across the thorax and epigastrium.

"I was called in about five o'clock, when the preceding details were given me. He described the anguish as still very keen not only in the body but in the arm; yet he appeared to respire without much difficulty. He had excessive flatulence, and experienced slight relief from frequent eructation. His pulse was 90 and not hard, and his tongue was clean.

"I prescribed for him some calomel and opium in conjunction with opening medicine, and at half-past nine o'clock was informed that he was more easy. His friend remained in the room with him till one o'clock, and then left him still better. At four the servant went into his chamber, when he reported greater amendment; and at six his niece visited him, when he felt so well as to talk of going into the country by the morning coach, but, on being told that it snowed, he consented to defer his departure till I had seen him. At eight o'clock he gave instruction as to his breakfast, sat up in bed, conversed cheerfully and energetically with his nephew, spoke of his relief with gratitude to the Almighty, when suddenly he complained of faintness, fell back on the bed, and instantly expired."

Such is the treachery and uncertainty of this terrible disease! The victim is generally in least danger when apparently in the jaws of death—and suddenly expires, when free from suffering.*

Mr. Cooke examined the body in the evening of the day he died. The spleen was three or four times its natural size—there were some appearances of inflammation and thickening in the mucous membrane of the stomach—lungs healthy—an ounce of bloody serum in the cavity of the pericardium—"heart remarkably flaccid, and its fibres easily lacerable." The lining membrane of the chambers was unusually red, and, on the mitral and semilunar valves, were many opaque spots—"but, in no part, had ossification taken place." "*Arteritis* had existed to some extent up the aorta, and the posterior surface of the valves was more diseased than other parts." There was no appearance of disease in the coronary arteries, "except their orifices being involved in the morbid process going on in the aorta." Mr. Cooke observes that he has seen some other cases where the pathological characters were the same. This case and these observations tend to confirm the doctrine that a flabby state of the heart (very probably dependent on an affection of the nerves of the organ) is the most common of all post-mortem appearances. Mr. Cooke is, no doubt, well aware of the deception attendant on redness of the lining membrane of the heart and arteries. He did not, of course, pronounce that there was actual *arteritis*, without being fully convinced that he was not led into error by changes that take place after death.

---

### 9. Function of the Liver—Use of the Bile.

In Mr. Abernethy's lecture on this subject, there are some curious opinions delivered. Dr. Powell told him—and he is himself convinced that, "there is no peculiar matter in the bile which gives it its bitterness and colouring." Mr. A. thinks they must depend "on some arrangement which we do not understand." He has not made up his mind whether the liver be an organ for separating something noxious from the blood—or elaborating something necessary for chylifaction. It is evident, however, that he does not place much faith in the chylifactive properties of bile. "I see patients, says he, whose biliary discharges have been suspended for months, and yet they seem to be well nourished." Now, if there is any thing better established by observation than another, it is the fact that suspension or vitiation of the bile produces a rapid emaciation of the body. In respect to sympathy, Mr. A. believes that disorders of the head will affect the stomach, and *vice versâ*. In illustration of the influence of irritation of the bowels Mr. A. relates rather a dirty story of a London doctor coming home from a London Tavern dinner—and feeling very uncomfortable, being obliged to limp and creep over the ground. Having visited the Temple of Cloacina, on his way back, he got eased of such a cargo, that he "jumped and walked home with boyish alacrity." We do not see very clearly how this exemplifies the influence of intestinal irritation on the *mind*. It shews indeed, that it will affect the nervous and muscular powers of the body; but the lecturer might have selected numerous and interesting illustrations of this said irritation on the very highest faculties of the intellect—the judgment, the memory, the imagination, &c.

Mr. A. adverts to the influence of the hepatic functions on the mind, and repeats the old story of *hypochondriasis* being owing to "something wrong in the *hypochondrium*." What that something is, the lecturer does not say—but infers that it is the duty of medical men to "keep the functions of the liver as right as they can." It would be desirable to know *how* this is to be done! We apprehend that the perpetual reiteration of the blue-pill and black draught, is not the very best method.

In treating of organic diseases of the liver, the facetious lecturer observes "here I get over the ground rather lightly, for there are certain organs in the body which, if the vessels go into a state of diseased action, they seem to me to produce but one, or scarcely any thing else but one kind of morbid structure. It is an infusion of something into the interstitial parts, in larger or smaller masses, and this we call tubercles."

---

* See the case of General B———, in No. 16 of this Journal, page 431.

Unquestionably this is getting over the ground of pathology lightly enough !

On the question whether hydatids be animalcula or merely coagulable lymph, Mr. A. gives the weight of his opinion to the former doctrine. It would appear that Mr. A. was a good deal influenced in his determination on this knotty point, by the following occurrence. A sheep died of apoplexy, and when Mr. A. opened the head, " out has *leaped* one of those globular hydatids." To deny an independent existence to *bodies* endued with such *saltant* propensities, and that without any legs or arms, would be unreasonable in the extreme. " If you *catch* one of them in this way, in warm water, you would undoubtedly think that there is vitality in it." We would advise Mr. A. to peruse the curious work of Rhudolphi on the subject of hydatids, and he will be convinced that what he only conjectures, has been proved over and over again many many years ago—long indeed before Mr. A. was born.

Mr. A's observations on gall-stones are extremely meagre. He says " there seems to be some law of crystallization prevailing among them." He thinks that very large ones sometimes make their way from the gall-bladder into the colon, by ulceration. This may be true ; but Mr. A. ought to have known that the ducts themselves have become ulcerated, and that gall-stones have made their way into the duodenum—where they have also produced obstruction, and, in a very few instances, death.

---

### 10. AMPUTATION DURING GANGRENOUS INFLAMMATION.

Notwithstanding the recommendation of Baron Larrey, the surgical profession seems very averse to have recourse to the ULTIMUM REMEDIUM of amputation, while gangrene is advancing towards the patient's body. Still, in desperate cases, it is allowable to make use of desperate remedies. The following affords a ray of hope in such emergencies.

A private soldier, 22 years of age, of healthy constitution and sober habits, fell from the window of a convent (what he was doing there is not said) upwards of 30 feet high, (at Lima in Portugal) and dislocated his right shoulder, at the same time fracturing both bones of his right leg, four inches above the ankle-joint. The fracture was compound, but the wound was small. Considerable depression was the immediate consequence of the accident and the fright. The fracture was easily reduced during the flaccid state of the muscles, but the leg was found to be much swelled, and much effusion of blood had taken place under the integuments, which were detached about two inches from the bone. On the day after the accident, the leg was still more swelled, without any increase of temperature, symptomatic fever, or local inflammation. The third and fourth days passed without any unfavourable symptoms ; but, on the 5th day, the leg became swelled, œdematous, and of a deadly pale colour, with a burning heat at the instep. In the night of that day, " a most rapid and disastrous change took place. The sixth morning presented the fore-part of the leg from the knee to the instep, in a state of gangrene ; while the parts in the neighbourhood of the fracture were in that of complete sphacelus. The disease ascended rapidly along the limb, and had now gained the middle of the thigh, the stomach being irritable, the countenance sunk, pulse low and weak. Under these circumstances, Mr. Macdermott, the surgeon of the regiment, amputated about three inches below the trochanter major, " the integuments even there being discoloured and œdematous." The symptoms after the operation were formidable, but the patient rallied, under laudanum and brandy, and although there was a good deal of sloughing of the cellular membrane about the stump, the man recovered, and the stump healed.

It was found that a splinter of bone, three inches long, had been completely detached from the tibia, longitudinally, and forced down between the lower portion of that bone and the fibula. Mr. M. considers this case as affording " a strong proof of the propriety of amputating without waiting for a line of demarcation to be formed, when the progress of mortification, as in this instance, threatened not only the loss of limb, but of life also."—*Ed. Journ. April.*

---

### 11. BURNS AND SCALDS.

The subject of burns and scalds excited a pretty *hot* discussion in the Westminster Medical Society during a late sitting, in consequence of a paper read by Mr. Bingham. We shall not, on this occasion, follow either the writer of the Essay or the orators who delivered their opinions on particular points of theory or practice; but we shall give a brief summary of the general sentiments that appeared to prevail on the subject under consideration. Oral discussions in medical societies have one advantage over written documents. The latter set forth the opinions or practice of an individual—or a compilation from books written perhaps ages before. In Medical Societies, we hear the sentiments of many men fresh from clinical practice, and who have not had time or temptation to work up their observations into a system, or strain their brains in the formation of an hypothesis. The papers brought before the Westminster Medical Society are, in general, much too long. They embrace etiology, symptomatology, pathology, and therapeutics—and the reading of a paper, nine-tenths of which are merely copied from the class-books of the schools, occupies one of the two hours dedicated to the meeting of the members. Insulated cases, or single points in pathology or practice generally elicit far more information from the speakers than these systematic THESES.

Burns and scalds may be conveniently enough divided into those which merely excite the skin, or produce vesications—and those which inflame or disorganize deeper seated structures, down to the bone itself. In all severe or extensive burns, however, there are some parts in one of these conditions, and some in the other. Burns may produce only effects on the parts burnt—or they may produce a shock *immediately* on the nervous system, resembling that which succeeds a concussion of the brain. It is evident that, in such cases, we must give cordials, stimulants, laudanum, till some re-action takes place, when we are to be very guarded in the use of these means. The excitement of the system—the fever, in fact, which follows some time after the infliction of the injury, is quite another thing, and must be treated like all other sympathetic excitements of the system. In respect to local applications, are we to go on the stimulating plan of Kentish, or the cooling plan of other authors? Are we to apply refrigerating, that is, evaporating lotions—or oil of turpentine and linseed oil. There is less difference in these applications, perhaps, than is generally imagined. If a finger be burnt, by touching a hot poker, or by being sprinkled with boiling fluids, and we plunge it in cold water or snow, the burning sensation is instantly soothed, for a time, till the temperature of the application rises a little, and then we must plunge it afresh into colder materials. On the other hand, let the part be wetted and held near the fire;—the sensations are rendered, for a few minutes, much more pungent; but, after a little time, the pain is almost entirely obliterated—we mean, of course, in superficial burns. This illustrates we think, the operation of oil of turpentine and other stimulating applications to burns, both in the superficial and the deep-seated degrees. The experience of the Society—at least of those who, from local circumstances, had most experience, was in favour of Dr. Kentish's plan to all kinds of burns and scalds. Dr. A. T. Thompson advocated the beneficial effects of combining opium in solution with spirituous applications. Messrs. Skene and Greene, who had practised in coal mine countries, where explosions of hydrogen gas produce such terrible consequences, gave their testimony to the stimulant practice—combined with opium and cordials internally. Purgatives were found to be very beneficial in severe burns. The author of the paper had taken no notice of the effects produced on internal organs, in severe injuries of this kind. It was shewn that inflammation of the mucous membrane of the stomach and bowels—effusions into the cavities of the pleura and brain, &c. were often the immediate causes of death in those cases which terminated fatally, some days after the first shock was over.

A gentleman detailed some cases from Bartholomew's Hospital, where the application of cotton, according to the plan reported by Mr. Plymsoll in a late Fasciculus of this Journal, was eminently beneficial. We understand the practice is now undergoing a fair trial in that hospital, and the results will, no doubt, be soon made known.

12. Treatment of Syphilis without Mercury.

The modern mode of treating syphilis without mercury, although supported, to a certain extent, by high authority, is not yet sufficiently established to render additional confirmation of its efficacy unnecessary. M. Fricke adopts a line of practice in the venereal cases which are admitted into the hospital at Hamburg, very nearly resembling that recommended by Thompson and other English practitioners. 1mo. Every patient is bled to the amount of from six to twelve ounces, and the operation is repeated if necessary. About half a drachm of sulphate of magnesia is then given every three hours, and continued until repeated evacuations are produced. If the bowels afterwards become constipated, or the *ulcers heal slowly*, the use of the same remedy is renewed. 2do. As an external application to the chancres, goulard water, or two grains of sulph. zinc, in six ounces of distilled water, is employed. When the size of the sore is much diminished, and it is no longer painful, lime water is used. If either of these lotions cause pain or inflammation, they are to be still farther diluted. Buboes are first treated by compression, and, if resolution cannot be promoted, they are opened with a bistoury, and afterwards dressed with dry lint. Condylomatous tumours are removed by the knife, or cauterized, and the wound dressed with the same lotion as for the chancres. 3tio. The patients are kept upon very low diet, consisting of vegetables, bread, and "soupe à l'eau," twice a day. 4to. If, at the end of a few days, the symptoms are not alleviated, a few doses of mercury, in small quantities, are given, and are found sufficient to effect a cure.

The results obtained by this mode of practice are highly satisfactory. Chancres and buboes are speedily cured, and the cicatrices are by no means so evident as when mercury has been employed. Chancres, from three to four lines in diameter, are generally cured, in female patients, in from one to three weeks. Rather a longer time is required in male patients. M. Fricke, who has the advantage of retaining under his observation the patients thus treated, has not yet observed any secondary symptoms.—*Graefe and Walther's Journ. der Chirurgie.*

13. New Method of treating Syphilitic Diseases.

M. Dzondi, Professor of Halle, administers the corrosive sublimate in the following manner.

℞. Deuto. chlor. hydrarg. gr. xij.; solve in aq. distill. q.s., et adde—

Micæ panis et sach. alb. āā q.s. ut ft. mass. pro pilul. 240.

On the first day, the patient takes four pills ($\frac{1}{5}$ of a grain.) The second day he takes none. The third, six pills. The fourth none. The fifth, eight pills, and so on. The twenty-seventh day, $1\frac{1}{2}$ grain will thus be taken, and at this time, in a great majority of cases, the cure is complete. During the treatment, decoct. sarsæ is taken as a drink, and the patient is restricted to half the quantity of food he was previously accustomed to take. Pork, game, cheese, milk, acid dishes, and spirituous liquors, are prohibited. He is only permitted to leave the house during very fine weather, and never either early in the morning or late in the evening. All external applications to local symptoms are to be carefully avoided. The change they undergo is to be the test of the action of the mercury upon the general system. The dose of the mercury is to be increased until some evident amelioration is perceived. Whatever may be the quantity, it is to be given at one time, and immediately after dinner. If colic or diarrhœa should be provoked, a few drops of laudanum are to be given. Besides the advantage which this method possesses, of effecting a radical cure, it does not cause salivation, is economical, and speedy. It rarely happens that the treatment lasts longer than a month. Dzondi assures us that he has cured all his syphilitic patients in this manner for the last ten years.—*Neue Zuverlaessige Heilart der Lustseuche in allen ihren formen. Halle*, 1826.
